# Antagonistic Interactions between the African Weaver Ant *Oecophylla longinoda* and the Parasitoid *Anagyrus pseudococci* Potentially Limits Suppression of the Invasive Mealybug *Rastrococcus iceryoides*

**DOI:** 10.3390/insects7010001

**Published:** 2015-12-23

**Authors:** Chrysantus M. Tanga, Sunday Ekesi, Prem Govender, Peterson W. Nderitu, Samira A. Mohamed

**Affiliations:** 1 International Centre of Insect Physiology and Ecology (icipe), P.O. Box 30772, Nairobi 00100, Kenya; ctanga@icipe.org (C.M.T.); pnderitu@icipe.org (P.W.N.); sfaris@icipe.org (S.A.M.); 2 Department of Zoology and Entomology, University of Pretoria, Pretoria 0002, South Africa; prem.govender@smu.ac.za; 3 Faculty of Health Sciences, Sefako Makgatho Health Sciences University (SMU), P.O. Box 163, Ga-Rankuwa 0221, South Africa

**Keywords:** *Oecophylla longinoda*, *Anagyrus pseudococci*, *Rastrococcus iceryoides*, mummy mutilation, parasitoid mortality, biotic interference

## Abstract

The ant *Oecophylla longinoda* Latreille forms a trophobiotic relationship with the invasive mealybug *Rastrococus iceryoides* Green and promotes the latter’s infestations to unacceptable levels in the presence of their natural enemies. In this regard, the antagonistic interactions between the ant and the parasitoid *Anagyrus pseudococci* Girault were assessed under laboratory conditions. The percentage of parasitism of *R. iceryoides* by *A. pseudococci* was significantly higher on “ant-excluded” treatments (86.6% ± 1.27%) compared to “ant-tended” treatments (51.4% ± 4.13%). The low female-biased sex-ratio observed in the “ant-tended” treatment can be attributed to ants’ interference during the oviposition phase, which disrupted parasitoids’ ability to fertilize eggs. The mean foraging time, host handling time and number of successful oviposition in “ant-excluded” treatment were significantly higher compared to “ant-tended” treatments. When ant workers were allowed access to sterilized sand grains, mummified and unmummified *R. iceryoides*, they selectively removed the mummified mealybugs, indicating that they recognized the mummies as potential foods (1.2 ± 0.46 to 7.8 ± 1.17 mummies at 10 min intervals for 2 h). Percentage emergence from mummified *R. iceryoides* removed by the ants was significantly lower compared to emergence from mummies not exposed to ants. Although, host seeking parasitoids frequently evaded attacks, some were killed by the foraging ant workers (2.0 ± 0.38 to 6.0 ± 0.88 at 10 min intervals for 2 h). These results suggest for the first time that the presence of *O. longinoda* has a detrimental effect on the abundance, reproductive success and possibly oviposition strategy of female parasitoids, which might be a delimiting factor in field conditions if both natural enemies are to be recommended for use within the same agro-ecosystem.

## 1. Introduction

*Rastrococcus iceryoides* Green (Hemiptera: Pseudococcidae) was inadvertently introduced into Tanzania, Kenya and Malawi in the early 90s, where it is potentially posing a serious threat especially on mango production [1,2,3,4]. In its native range of Southern Asia, *R. iceryoides* is believed to be highly polyphagous compared to *R. invadens*, which was first detected in 1982 and spread rapidly invading the coastal regions of most West and Central African states causing overwhelming losses of 50%–90% of mango, coupled with social and cultural problems [5]. The host range of *R. invadens* has been well documented with 45 plant species from 22 families classified as forest trees, vegetables, shade trees, fruit trees and ornamental plants [6] compared to more than 80 known host plants from 35 families in Southeast Asia [1,7] and 29 host plants from 16 families in Kenya and Tanzania for *R. iceryoides* [4]. However, *R. invadens* was brought under control in West and Central African states by release of two exotic encyrtid wasps, *Gyranusoides tebygi* Noyes and *Anagyrus mangicola* Noyes, from India [5,8].

The feeding behaviour of *R. iceryoides* on their associated host plants causes premature leaf drops, shedding of inflorescences and fruit-lets, as well as severe twig dieback effect [4,9] *Rastrococcus* *iceryoides* has a high fecundity with nine to ten generations per year, and excretes abundant honeydew on which sooty mold develops [4,10], thus reducing fruit marketability. As a result of the development of sooty mould, export opportunities are often impaired due to quarantine regulations [11]. In Tanzania, Kenya and Malawi, mango losses due to this pest can range from 30% to complete crop failure in unmanaged orchards [3,4]. Since its accidental introduction into Africa in the early 1990s [1,2], management has relied largely on repeated applications of insecticides, but the use of chemical insecticides are not always effective for the management of several species of mealybug due to the heavy layers of waxy coating that shield their body [12]. The egg stage of *R. iceryoides* in particular and several other mealybug species are protected by a thick waxy ovisac [4,13], which most insecticides cannot penetrate [14]. This combined with the extremely wide host range makes it almost impossible to have a spraying program capable of bearing the cost and coping with the practicalities of treating the whole range of infested plants in an affected area [15]. As a result, the most common method used by some growers is cutting down of heavily infested mango trees, while others have abandoned mango cultivation altogether.

Therefore, as an alien invasive pest species in Africa, *R. iceryoides* is an obvious target for classical biological control. The exploration for natural enemies of *R. iceryoides* in its putative home of origin is currently being undertaken by the African Fruit Program (AFFP) under the umbrella of the International Centre of Insect Physiology and Ecology (*icipe*) and this will be immediately followed by importation of promising species to Africa for evaluation and ultimate release. However, before embarking on the introduction of coevolved natural enemies from the indigenous home of the pest, the composition as well as the efficacy of indigenous natural enemies, which may have formed new associations with this pest in Kenya and Tanzania, was established [4,16]. These studies revealed that *Anagyrus pseudococci* Girault (Hymenoptera: Encyrtidae) was the most widely distributed indigenous primary parasitoid of *R. iceryoides* in both countries accounting for 95% of the overall parasitism on the pest [4]. In the light of this knowledge, the potential use of *A. pseudococci* in augmentative biological control of *R. iceryoides* is currently under evaluation at *icipe*, Nairobi, Kenya.

On the other hand, the African weaver ants *Oecophylla longinoda* Latreille (Hymenoptera: Formicidae) are the most dominant and important ants in tree canopies of the humid tropics of Africa [17,18]. The mango mealybug also has a spatial refuge under tree canopies, where it is protected from extreme temperatures, natural enemies and insecticide applications [4]. Several studies have shown that ants tend honeydew-producing Hemipteran insects such as mealybugs to access a renewable and defensible source of carbohydrates energy-rich food [19]. In return, the ants render protection against parasitoids, predators and even their competitors [20,21], as well as sanitation [22]. The relative effectiveness of the Africa weaver ant *O. longinoda* in reducing the incidence of predation and parasitization in different Homoptera has rarely been examined. By providing protection to the mealybugs from natural enemies, the presence of certain ant species can be detrimental to the impacts of biological control [4,23,24]. It is important to note that the ultimate success of both indigenous and exotic biological control agent is crucial and depends largely on their relationships with resident species in the invaded area as well as many other environmental factors [25]. This implies that if there is any harmful biotic interference between resident organisms and the released biological control agent, then establishment and/or efficacy of that biological control agent can be affected negatively [26]. For example, Chacón *et al.* [25] has shown that biotic interference can be responsible for close to 20% of failures of most classical biological control programs.

The use of predatory African weaver ant *O.* *longinoda* has recently been advocated as a component for inclusion within mango fruit fly integrated pest management programs in Africa; as fruit damage can be reduced as a function of the ant abundance [18,27]. Several studies have shown that *O. longinoda* has the ability to significantly reduce pest damage in many tropical tree crops [20,28,29,30,31,32] and are in this way known to improve fruit quality [30,33]. Although ants are excellent natural predators and can be used as biological controls for some pests as described above, virtually all ant species that prey on insect pests also possess some potential disadvantages [34]. For example, recent reviews have demonstrated that *O. longinoda* and other ant species are relatively indiscriminant, omnivorous predators that attack beneficial insects in addition to pests and may play a key role in reducing the efficacy of some natural enemies in several agro-ecosystems [35,36,37,38]. *Oecophylla longinoda*, in particular, is highly aggressive and strongly predaceous, using their considerable strength and cooperative ability to capture a wide range of insect prey that venture onto their colonies, spread over large territories on canopies of host trees [39]. Ant foraging on plant canopies has been reported to significantly reduce natural enemy activities and promote mealybug infestation to a significant level [40]. The disruption of parasitoid species activities can be attributed to the differences in their behavioural avoidance of ants, length of oviposition period, and their ease of disturbance during oviposition [41,42].

Although most parasitoids are severely hampered by aggressive ant species, Way [43] pointed out that *O. longinoda* workers do not attack very small insects such as parasitoids (*Coccophagus* spp.) of their attended scale insect, *Saissetia zanzibarensis* William (Homoptera: Coccidae). Beside the observation by Way [43], no other information exists in the literature on the biotic interference likely to occur between other native parasitoids and the resident *O. longinoda*, to provide basic information for the development of reliable and cost-effective future management interventions of hemipteran pests in the presence of these ants. As part of an ongoing project on integrated pest management (IPM) of major mango pests, the purpose of this study was to examine under laboratory conditions (i) the effect of *O. longinoda* on the incidence of parasitism of *R. iceryoides* by *A. pseudococci* and parasitoid emergence; (ii) host-handling time and oviposition success of *A. pseudococci* in the presence and absence of *O. longinoda*; (iii) effect of *O. longinoda* abundance on parasitoid emergence based on close examination of mummified *R. iceryoides* removed by the ants; (iv) assess mortality inflicted by *O. longinoda* on the parasitoids and behavioural responses displayed by the parasitoid to evade encounters with workers of *O. longinoda*.

## 2. Experimental Section

### 2.1. Mealybug and Parasitoid Wasp Colonies

The colony was initiated from a cohort of 300 adult *R. iceryoides* that were collected from a mango tree in Matuga, (04°11'02.5'' S, 039°33'38.4'' E, 109 m above sea level), Coast Province of Kenya on the 13 February 2008 and transported to *icipe*, Nairobi, Kenya. In the laboratory at *icipe*, butternut squash were surface sterilized with 5% sodium hypochloride, followed by three rinses in sterile distilled water to prevent fungal growth and later air dried for 24 h. Thereafter, the surface sterilized butternuts were infested with the mealybugs collected from the field. For colony maintenance, bi-weekly infestation of 10–20 butternut squash was carried out, and after every six months, wild populations of *R. iceryoides* from the field were introduced to ensure a broader genetic diversity in the laboratory colonies. The mealybugs were maintained on butternut squash for approximately 20 generations to allow adaptation to their new host. Cultures were maintained at ambient conditions (26 ± 2 °C, 60%–70% relative humidity (RH), and photoperiod of 12 h L: 12 h D). Before the start of the experiments, mealybugs were collected from the stock colony and maintained until third instar nymphs, which are the most preferred developmental stage for oviposition by *A. pseudococci* [44]. The parasitoid colony was initiated from mummified *R. iceryoides*, collected from the same mango tree. The mummified mealybugs were kept in a well-ventilated transparent Perspex cage (30 cm length × 30 cm width × 30 cm height) with a moistened cotton ball in the laboratory at room temperature. The emerged parasitoids (72 females and 21 males) were allowed to mate for 48 h and then reared on third instar nymphs and adults of *R. iceryoides* maintained on butternut squash in a well-ventilated Perspex cage (30 cm length × 30 cm width × 30 cm height). The parasitoids were provided with fine drops of pure honey streaked on the top-side of the cages as food and moist cotton wool as water sources. The parasitoid colony was periodically refreshed every six months with field-collected insects and maintained at ambient conditions (26 ± 2 °C, 60%–70% RH and a 12:12 (L:D) photoperiod).

Before the start of the experiment, 50 adult wasps were collected from the stock colony weekly and released into another Perspex cage (30 cm length × 20 cm width × 20 cm heights) containing two butternuts, each infested with 500 third instar nymphs of *R. iceryoides.* Parasitoids were maintained on butternut squash for at least 10 generations (from egg to adults) to allow them to adapt and to remove maternal effects [45], thus minimizing the effects of associative learning during the experiments. At the start of the experiment, 12 days after the release of the parasitoids, mummies were collected from the butternut squash and individually placed in gelatin capsules, and newly emerged parasitoids were used for the experiments.

### 2.2. Ant Colonies

Several ant nests of *O. longinoda* containing major and minor workers together with queens were collected from a tree at Muhaka (04°19'24.8'' S, 039°31'35.3'' E, 30 m above sea level), Coast Province, Kenya on 14 February 2008. The ant nests were carefully transported to *icipe* and then placed on branches of potted *Ficus* *benjamina* L. (Family: Moraceae) seedlings in a screen-house (2.8 m length × 1.8 m width × 2.2 m height), since different colonies have been reported to be mutually antagonistic [39]. The potted *F. benjamina* plants were maintained on tables (245 cm length × 78 cm width × 75 cm height). The edges of the tables were smeared with Tanglefoot^®^ insect barrier paste (The Tanglefoot Company, Grand Rapids, MI, USA) while the feet of the table were placed in containers filled with soapy water to keep away predators. The *O. longinoda* colonies were provided with living insect food sources (for example, dipterans’ larvae and adults, termites, caterpillars, lepidopteran adults, grasshoppers, worms, *etc.*) three times a week. Studies have shown that *Oecophylla* ants fed exclusively with insects would result in highly aggressive ants [46]. Other high protein food sources, especially fish intestines, were also provided twice a week to augment the weaver ant population. Cotton wool balls soaked in diluted honey solution were also provided and replaced every 48 h. The rearing conditions at the screen house were maintained at 22.3 ± 5.07 °C, 40%–80% RH and 12 h L: 12 h D photoperiod.

### 2.3. Effect of O. longinoda on Parasitism of R. iceryoides by A. pseudococci and Parasitoid Sex Ratio

Butternut fruits infested with 100 third instar nymphs of *R. iceryoides* were used in this experiment. A total of 100 workers of *O. longinoda* were transferred into the cages holding the mealybug-infested butternut fruits and allowed to forage for 3 h. Thereafter, 10 three-day old female and 10 male wasps were aspirated from the rearing cages and gently introduced into the experimental cages together with food as described above. An ant-free cage with *R. iceryoides* and parasitoids only was used as “control”. After 24 h exposure, the ants and parasitoid wasps were removed. Cages with exposed *R. iceryoides* were then allowed to stand for 10 days, after which mummified *R. iceryoides* were recorded daily. When parasitoid emergence was completed, the number of the mummies with parasitoid exit holes and numbers of unenclosed mummies were recorded. The latter were then dissected under the microscope and their contents (dead mealybug, dead parasitoid, empty shell, or undifferentiated mass) were recorded. Sex ratio was calculated as the proportion of females to the total number of emerged wasps. The experiment was maintained at 26 ± 2 °C, 60%–70% RH with a 12 h: 12 h (L: D) photoperiod. The experiment was replicated 10 times.

### 2.4. Host Handling Time and Oviposition Success of A. pseudococci in the Presence and Absence of O. longinoda

Newly emerged wasps were sexed, mated and fed with pure honey and water as described above. In this experiment, only 72 h old host-deprived female wasps were used because high egg load is a major parameter of foraging motivation and oviposition pressure [16,47,48]. The experiment consisted of “ant-tended” and “ant-excluded” treatments with 100 adult *O. longinoda* workers used from each replicate of the treatments. Observation period for each treatment began when a single mated female wasp was introduced into the cage containing butternut infested with 50 unparasitized third instar nymphs of *R. iceryoides*. After the wasp was introduced into the experimental arena, qualitative descriptions of the time spent for each observational parameter was recorded. In this study, host-handling time is defined as the sum of adult parasitoid female examination of *R. iceryoides*, probing and drilling as well as oviposition time during the observation period in both “ant-tended” and “ant-excluded” treatments. Following each host encounter, host-handling was divided into three responses: (a) host examination—for *A. pseudococci*, when a foraging female wasp encountered a host, they exhibited a stereotypical host examination process before rotating her body to face away from the host, showing clearly that it had recognized the potential host; (b) ovipositor probing and drilling—occurs when the foraging female wasp flexed the tip of her abdomen such that the tip of the ovipositor touches the host body plus a fast and rhythmical insertion of ovipositor into the host and (c) oviposition—characterized by a pumping movement of the abdomen and ending up with a strong and jerky withdrawal of the ovipositor (*i.e.*, host haemolymph exudes from the oviposition puncture). The experiment was terminated when the parasitoid was caught by ants or after an hour.

During the process of host-handling, *R. iceryoides* often exhibited a defensive response, which included raising and shaking its abdomen violently and rapidly in order to throw the parasitoid off its body or pulling its sucking mouth part and moving backward. This behavioural tendency exhibited by the host usually led to host rejection without initiating probing, drilling or oviposition. Therefore, after oviposition was completed during each interactive phase, stung *R. iceryoides* (*i.e.*, mealybug bleed haemolymph) were circled with a permanent marker pen and allowed to continue feeding on the butternut for three days. From direct observation it cannot be determined whether each drilling bout made by the parasitoid resulted in oviposition or how many eggs were deposited. Therefore, the stung mealybugs from the different treatments (“ant-tended” and “ant-excluded” treatments) were dissected in phosphate buffer solution (PBS) using Leica MZ 125 Microscope (Leica Microsystems, Heerbrugg, Switzerland), fitted with Toshiba 3CCD camera. The parasitoid eggs were then observed using an Auto Montage software (Syncroscopy, Synoptics group, Cambridge, UK) fitted to the computer at magnification of X25. The number of parasitoid eggs oviposited in each mealybug was counted microscopically to determine oviposition success. Each *A. pseudococci* female was observed only once, with a total of 15 female wasps in each treatment.

### 2.5. Effect of O. longinoda Abundance on Parasitoid Emergence

Prior to the experiments, five different groups of *O. longinoda* (1, 5, 10, 15 and 20 ants) were fasted for 24 h in the laboratory. At the start of the experiment, the different ant groups were carefully introduced into their respective transparent Perspex cages (60 cm length × 60 cm width × 50 cm height) and allowed to interact for 30 min. The Perspex cages had a small opening by the side (2 cm in diameter) through which a cord (1 cm thick) linked it to another cage of similar size, 90 cm away from the experimental arena to represent the ant’s nest. The negative control consisted of 30 sterilized sand grains (approximately, 3 mm diameter) placed on top of carefully cut circular portions of the bottom of butternut fruits in Petri dishes (9 cm diameter). The positive control was a group of 30 unparasitized *R. iceryoides* randomly placed on circular cut portions of butternut, as described above, 24 h prior to the experiment. The test treatment comprised of 30, eight-day-old mummified *R. iceryoides* containing *A. pseudococci* immature instars also randomly placed on the circular cut portions of butternuts in Petri dishes. After, 30 min following the introduction of the ant groups into their respective cages, the Petri dishes containing mummified *R. iceryoides*, unparasitized *R. iceryoides* and sterilized sand grains were gently introduced to the center of the experimental arena of the ants and allowed an interaction time of 5 min. Thereafter, continuous observations were made at every 10 min interval for a total duration of 2 h to record the number of mummified *R. iceryoides*, unparasitized *R. iceryoides* and sand grains removed from the Petri dishes by the ant workers and transported successfully to the next cage connected with the cord as described above. The Petri dishes were randomly oriented at the start of each replicate in order to randomize the sequence of the discs. After each experiment, the ants were removed and the total number of mummified *R. iceryoides* transported over the 2 h observation period by the different ant groups was kept separately until emergence of the adult parasitoid wasps. Additional Petri dishes containing 30 mummified *R. iceryoides* not exposed to the ant workers were introduced as control for the adult parasitoid eclosion experiments. Emerged parasitoid wasps were counted and expressed as a percentage of the initial number of mummified *R. iceryoides* removed by the ant workers at the end of each experiment. The numbers of unenclosed mummies were recorded and dissected under the microscope as described above. Five replicates of this experiment were conducted for each cohort of ants.

### 2.6. Behavioural Experiment of Parasitoid Wasp

Before the start of the experiment, five butternut fruits infested with 200 third instar nymphs of *R. iceryoides* each were introduced into Perspex cages (90 cm length × 90 cm width × 60 cm height). Thereafter, 100 workers of *O. longinoda* that were fasted for 24 h were transferred into each cage and allowed to forage for 3 h before 30 three-day old mated *A. pseudococci* were introduced. Observations were made 10 min after the release of the parasitoid whereby the number of ants on the whole butternut and number of host-seeking parasitoids injured or killed by *O. longinoda* were recorded at 10 min intervals for 2 h. Additional cages containing 30 three-day mated *A. pseudococci* not exposed to the ant workers were used as “control” for the adult parasitoid mortality experiment.

Responses of adult *A. pseudococci* toward *O. longinoda* workers were classified into the following four behaviours: (1) fly away—in which the female flee away from the infested butternut on encounter with the ant; (2) jump away—on encounter with the ant without leaving the infested butternut; (3) change of walking direction—to avoid physical contact with the approaching ants; and (4) ignoring—the wasp continued its activities although at close contact with the ant. Observations were made at 10 min intervals for a total duration of 2 h to record the number of parasitoids that showed each behavioural response. Dead parasitoids were replaced immediately to make sure that same number of parasitoids was observed at each time throughout the 2 h observation period. This experiment was replicated five times.

### 2.7. Statistical Analysis

Data on percentage of parasitism, percentage of emergence, sex ratio, number of ovipositor penetrations of host, successful and unsuccessful oviposition, and the duration of behavioural sequences (searching time and host handling time) in both “ant-tended” and “ant-excluded” treatments were subjected to Student’s *t*-test. The percentage of parasitoid emergence after allowing access of mummified *R. iceryoides* containing immature stages of *A. pseudococci* to the different ant treatments were subjected to a one-way analysis of variance (ANOVA). The number of mummified *R. iceryoides* removed by different *O. longinoda* groups, the number of dead adult parasitoids killed by *O. longinoda* and behavioural responses displayed by the parasitoid to evade attack by *O. longinoda* at different time intervals were analyzed using a repeated measures analysis of variance. Data on percentages obtained during the study were arcsine transformed to comply with homogeneity of variance and normality assumptions before subjecting them to the various statistical analyses described above. Means were separated using Student Neuman-Keuls test and the alpha level was *p* < 0.05 to indicate significance. The analyses were implemented using R 2.13.1 [49].

## 3. Results

### 3.1. Effect of O. longinoda on Parasitism of R. iceryoides by A. pseudococci, Parasitoid Emergence and Parasitoid Sex Ratio

The percentage parasitism of *R. iceryoides* by *A. pseudococci* in the “ant-excluded” treatment was significantly higher compared to the “ant-tended” treatment (Table 1). The percentage emergence of *A. pseudococci* was also significantly higher in “ant-excluded” treatments compared to “ant-tended” treatments. The offspring sex ratio was female-biased and significantly different between the “ant-excluded” and “ant-tended” treatments (Table 1).

**Table 1:** Mean (±SE) percentage parasitism, adult parasitoid eclosion and sex ratio of *Anagyrus* *pseudococci* after 24 h exposure period to third instar nymphs of *Rastrococcus iceryoides* in the presence and absence of *O. longinoda*.

Parameters	Treatment		*t*-Test		
	Ant-Tended	Ant-Excluded	*t*	*df*	*P*
Parasitized Nymphs (%)	51.4 ± 4.13 ^b^	86.6 ± 1.31 ^a^	8.45	18	<0.0001
Adult parasitoid eclosion (%)	85.42 ± 2.72 ^b^	94.54 ± 0.55 ^a^	3.34	18	0.0069
Sex ratio (%)	62.2 ± 3.28 ^b^	71.67 ± 1.71 ^a^	2.54	18	0.0204

Within the same rows means followed by different letter are significantly different at *p* ≤ 0.05.

### 3.2. Host-Handling Time and Oviposition Success of A. pseudococci in the Presence and Absence of O. longinoda

The average host searching time by *A. pseudococci* on “ant-tended” experimental arena (treatment) was significantly longer compared to the “ant-excluded” treatments. Upon contact with the host, *A. pseudococci* required significantly longer time to complete all sequences of host examination and oviposition activities on a single host in “ant-excluded” treatment and in “ant-tended” treatment (Table 2).

**Table 2:** Mean (±SEM) host searching time, host handling time, ovipositor penetration and successful oviposition per h, when Anagyrus pseudococci females foraged in the presence or absence of Oecophylla longinoda.

Parameters	Treatment		*t*-Test		
	Ant-Tended	Ant-Excluded	*t*	*df*	*P*
Searching time (seconds)	440.9 ± 28.42 ^a^	334.5 ± 25.83 ^b^	2.77	38	0.0086
Host handling time (seconds)	71.07 ± 1.68 ^b^	79.53 ± 2.56 ^a^	2.72	28	0.0112
No. of ovipositor penetration/h	5.87 ± 0.72 ^b^	11.27 ± 1.29 ^a^	7.18	28	0.0093
No. of successful oviposition/h	4.13 ± 0.70 ^b^	9.87 ± 1.25 ^a^	4.02	28	0.0006

Within the same rows means followed by different letter are significantly different at *p* ≤ 0.05.

Dissection of exposed *R. iceryoides* from “ant-tended” and “ant-excluded” treatments showed that *A. pseudococci* deposited a single egg with each oviposition bout. However, it was not uncommon for *A. pseudococci* to drill in different locations on the same host. *Anagyrus pseudococci* achieved a significantly higher number of successful ovipositions in “ant-excluded” treatments compared to “ant-tended” treatments (Table 2).

### 3.3. Effect of O. longinoda Abundance on Parasitoid Emergence

**Figure 1:**
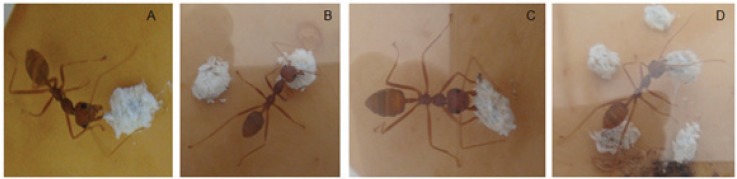
*Oecophylla longinoda* worker encounters with mummified mealybugs were always followed by an antennal examination (**A**) before transportation (**B**–**D**), indicating that they perceived the mummies as a potential food source.

**Figure 2:**
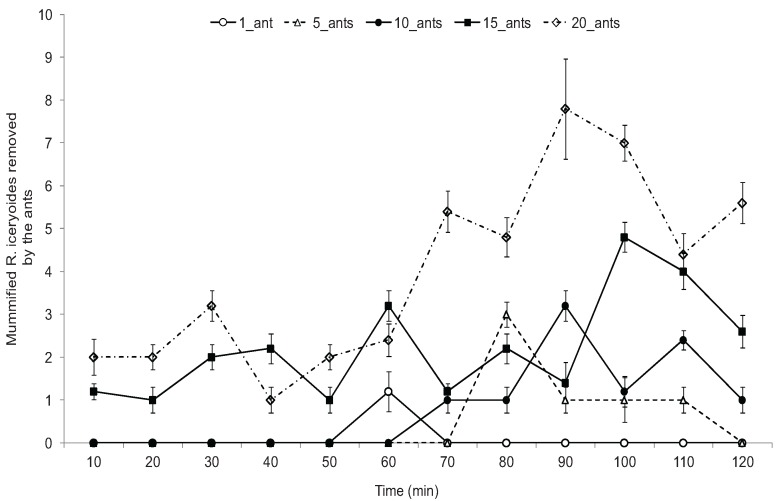
Mean number (±standard error) of mummified *Rastrococcus iceryoides* removed and transported by the different *Oecophylla longinoda* ant groups (treatments) from the foraging cages at 10 min intervals over a 2 h observation period.

When ant workers were allowed access to sterilized sand grains, mummified and unmummified mealybugs, they selectively removed the mummified mealybugs. Although, the different ant-groups were occasionally observed to perform antennal examination of the sand grains and unmummified mealybugs, none of these was transported out of the experimental arena to the next cage (90 cm away). When comparing the number of mummies removed across the different time intervals by the different ant groups, the 20 ant-group removed and transported a significantly higher number of mummies compared to its counterparts except at the intervals of 40 and 60 min. The interaction between the different ant-groups and number of mummified *R. iceryoides* transported by the ants over the 2 h observation period was significantly different (*F* = 8.13; df = 33, 396; *p* < 0.0001). No mummified mealybugs was removed by the 1 and 5 ant-group treatment within the first 50 and 70 min of observation, respectively. The number of mummified mealybugs removed at the 90th min (*F* = 342.46; df = 4, 182; *p* < 0.0001) and 100th (*F* = 179.87; df = 4, 98; *p* = 0.0038) by the 20 ant-group was significantly higher compared to the other ant-group treatments. Ant encounters with mummified *R. iceryoides* were always followed by an antennal examination before the ant workers finally transported the mummies away (Figure 1). During the 2 h observation period, the mean number of mummified *R. iceryoides* removed and transported by the different ant groups was observed to increase with time (Figure 2). There was a significant difference in the number of mummified *R. iceryoides* removed by the worker ants when compared across the different periods of observation (*F* = 2.79; df = 44, 220; *p* = 0.0104).

**Figure 3:**
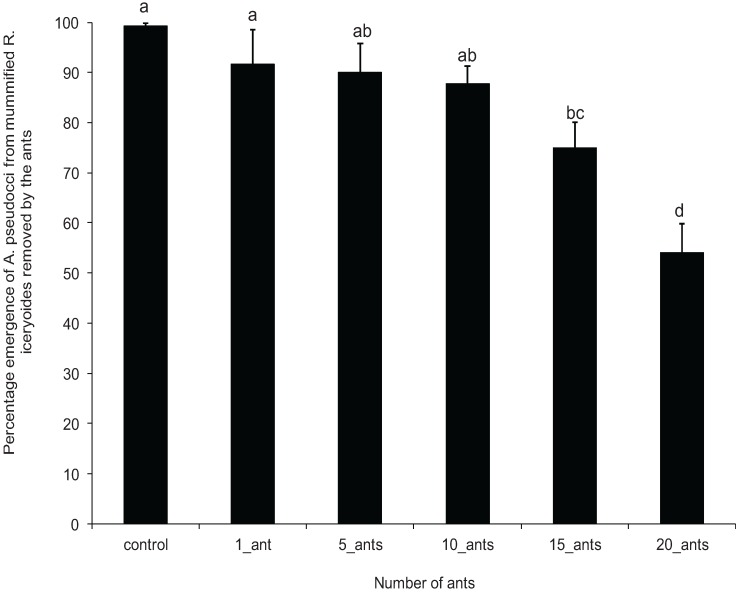
Mean (±standard error) percentage of *Anagyrus pseudococci* emerged from mummified *Rastrococcus iceryoides* removed by the different *Oecophylla longinoda* groups for a period of 2 h. Means with different letters are significantly different at *p* ≤ 0.05.

Mummified mealybugs removed by the single ant treatment had less mutilation as evident by a high percentage emergence of adult *A. pseudococci* (93.33% ± 6.29%) compared to that for the 20 ant-group treatment (54.09% ± 5.78%) (Figure 3). There was a significant difference in the percentage emergence of parasitoids among the different ant groups (*F* = 7.38; df = 4, 124; *p* < 0.0001). No significant differences of percentage emergence were observed between 1, 5 and 10 ant-group treatments compared to the control (Figure 3).

### 3.4. Mortality of A. pseudococci by O. longinoda and Parasitoid Behaviour Displayed to Evade Encounters

**Figure 4:**
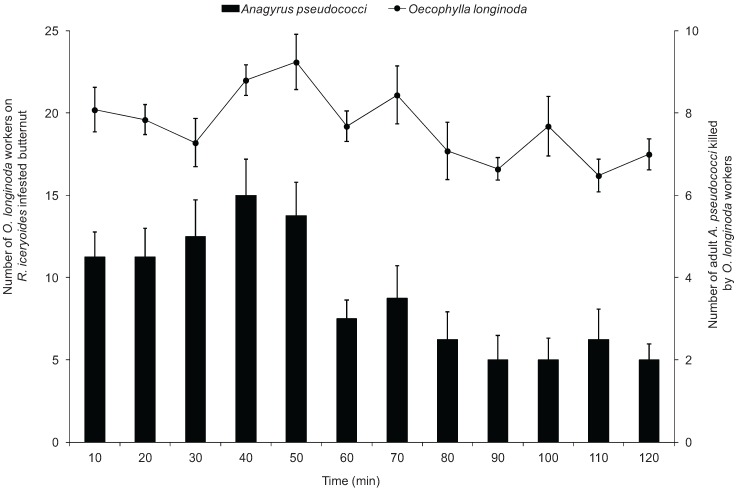
Mean (±standard error) number of *Oecophylla longinoda* on *Rastrococcus iceryoides*-infested butternuts and mean number of adult *Anagyrus pseudococci* killed by *Oecophylla longinoda* workers in the foraging cages at 10 min intervals over a 2 h observation period.

**Figure 5:**
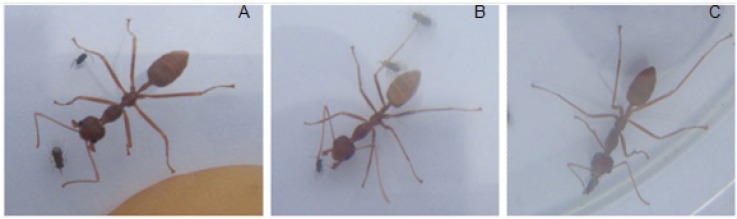
*Oecophylla longinoda* worker aggressively targets a fast running parasitoid (**A**); destabilizes it using its antenna (**B**) and finally seized the wasp with its mandibles (**C**).

The mean number of *O. longinoda* on *R. iceryoides*-infested butternut over the 2 h observation period ranged from 16.2 ± 1.0 to 23.1 ± 1.68 (Figure 4). Encounters between the ants and the host-seeking parasitoids were random rather than oriented search by the ants. The ants were frequently observed to exhibit aggressive behaviour towards *A. pseudococci* at every encounter. When confronted with the parasitoid, the ant workers detected them from a distance, opened their mandibles, and seized the parasitoid rapidly by a rapid forward movement of the whole body (Figure 5). Sometimes, *O. longinoda* workers were observed to exhibit reserved behavioural traits by walking more speedily and fluidly, allowing them to capture ovipositing female parasitoids. When the host-seeking *A. pseudococci* was successfully seized by *O. longinoda* workers using their mandibles, the parasitoids were sometimes released and picked up again immediately by the same or other ant workers. This repeated capture and recapture of the same parasitoids resulted in serious injury and sometimes eventual death of the parasitoid. The ant workers were found to remain still with captured parasitoids for some time before transporting them away (Figure 5). The highest number of parasitoids was killed at the 40th min and the lowest at the 120th min (Figure 4). There was a significant difference in the number of dead parasitoids caused by the ants over the 2 h period (*F* = 423.79; df = 1, 349; *p* < 0.0001) compared to the control. Figure 4 illustrates the trend in the number of parasitoids killed in the presence of ants over the 2 h period. The results suggest a highly significant interaction of parasitoids killed with time (*F* = 11.64; df = 9, 119; *p* = 0.0053).

**Figure 6:**
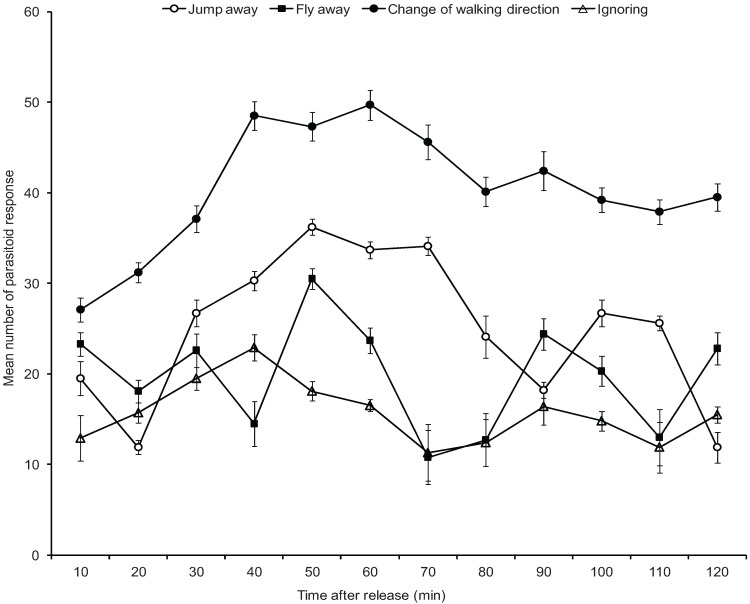
The mean (±standard error) number of behavioural responses displayed by the parasitoid *Anagyrus pseudococci* to evade encounters and attacks by *Oecophylla longinoda* at 10 min intervals over a 2 h observation period.

However, *O. longinoda* workers sometimes failed to seize the parasitoid wasps because they efficiently escaped from the ants’ grip during encounters or because the ant workers avoided or seemed not to be interested in the parasitoid. Escape behavior of the parasitoid wasps consisted of either jumping away with the highest response achieved at the 50th min (36.2 ± 0.89 observations) and to continue searching elsewhere on the *R. iceryoides*-infested butternut or flew away (highest values observed at the 50th min with 30.5 ± 1.12 observations) from the test arena in a direction putting them as far as possible from the ants (Figure 6). Ovipositing *A. pseudococci* were often observed to ignore approaching ants especially at the 40th min (22.9 ± 1.44 observations) and continued with the egg laying (Figure 6). The most common tactic used by the parasitoid to evade encounters with approaching ant workers was by retreating or repeatedly changing their walking direction (48.5 ± 1.59 and 49.7 ± 1.65 observations at the 40th and 60th min, respectively). The behavioural responses displayed by *A. pseudococci* on encounter with approaching ant workers varied significantly over the 2 h observation period (*F* = 8.13; df = 33, 396; *p* < 0.0001).

## 4. Discussion

*Rastrococcus iceryoides* is the most important mealybug pest of mango in East Africa [1,3,4], and *A. pseudococci* is the only major parasitoid we have routinely recovered from this invasive pest in Kenya and Tanzania [4]. *Anagyrus pseudococci* is thus of potential importance not only in the natural regulation of this pest but as a candidate for biological control programs that may be contemplated in the future. Although the efficiency of *A. pseudococci* in decreasing populations of several other mealybug species on their host plants has been well documented [50,51,52,53,54,55], this is the first comparable study with the mango mealybug *R. iceryoides*.

The offspring sex ratio was more female-biased in the “ant-excluded” treatment compared to the “ant-tended” treatments. Casual observations showed that sometimes there were physical contacts between the parasitoids seeking to utilize the host and the foraging ants. These interactions interrupted the parasitoids during ovipositor probing and drilling. The behavioural response of the parasitoid in the presence of the ant could delay the flow of spermatozoa released from the spermatheca during the fertilization phase, thereby increasing the production of unfertilized eggs. Therefore, the production of less female biased sex ratio in the presence of the ants may be attributed to the frequency of physical interference at the ovipositing site, as observed in the present study. Additional tests would be required to evaluate the role of these tactile interferences in the mechanisms by which female parasitoids change their offspring sex ratio in response to the presence of ants in a host-patch. In accordance to our study, findings by Boulton *et al.* [56] have also provided evidence to suggest that interference by male parasitoids (mating and/or harassment) during oviposition phase can completely disrupt facultative sex allocation, resulting in a less female-biased sex ratio because of female parasitoid inability to fertilize eggs. This is also similar to what was observed in *Caraphractus* *cinctus* (Walter) (Hymenoptera: Mymaridae) and *Nasonia vitripennis* (Hymenoptera: Pteromalidae), with the production of more male offsprings in the progeny in response to the frequency of physical and/or olfactory interference with other female (s) at the ovipositing sites [57,58]. Our findings have major implications for field releases of the parasitoids, since interference by the ants during the oviposition phase strongly influences the production of a more male-biased sex ratio.

In the present study, there is evidence of acceptability and suitability of *R. iceryoides* as host by the parasitoid *A. pseudococci*. This can be explained in part by the remarkable high level of parasitism (87%) and emergence (95%) achieved in the absence of the ants. This high level of parasitism and emergence can be attributed to the physiological compatibility between *R. iceryoides* and *A. pseudococci* [16], as the parasitoid does seem to share an evolutionary history with *R. iceryoides* (of Asian origin). The high percentage of parasitism observed in this study can also be attributed to short host-handling time by the parasitoid, which is beneficial because it increases the probability that the host will not escape before the egg is deposited. The efficiency of parasitism by *A. pseudococci* in the presence of the ants was found to diminish, although the parasitoid was able to gain access to the ant-protected resources and oviposit successfully in *R. iceryoides* by moving swiftly and avoiding encounters with approaching ants. This is in agreement with other studies that have noted a strong correlation between rapid host-handling time by *A. pseudococci* [37,59] or other parasitoid species with reduced success on ant-attended treatments [43,60]. This is further supported by several other studies, which point out that the activities of natural enemies of mealybugs are often disrupted by some species of tending ants, compromising the parasitization potential of mealybugs’ natural enemies and inducing further outbreaks of these economically important pests [36,37,61]. The mutualistic relationship between some ants and mealybug species is linked to the mealybugs’ honeydew, which constitutes an important food resource for ants, implying that the latter are capable of employing strong territorial defences and aggressive tendencies that might end up disrupting or killing parasitoids and/or predators just to protect the mealybugs [4,62].

The number of mummified mealybugs removed during the experiment was observed to increase with an increase in the number of ant workers. This implies that the ants might be capable of causing a significant reduction in the parasitoid’s immature life-stages. It is important to note that following antennal examination, ant workers were able to discriminate and selectively remove mummified *R. iceryoides* among unparasitized mealybugs and sterile sand grains. The role of the antennae in food finding by *O. longinoda* has been comprehensively described by Hölldobler and Wilson [39], which involves an elaborate tactile display as observed in our study. The worker ants transporting the mummified *R. iceryoides* were always trailed around by several other workers that finally come together, and on more than one occasion, they were observed pulling apart and dismantling mummies with their mandibles. The mutilation of mummified *R. iceryoides* by the ant workers is primarily a direct relationship resulting from the ants’ interest in associated mummified *R. iceryoides*. When the mummified *R. iceryoides* removed by the ant workers were incubated, the percentage of adult eclosion was significantly reduced due to the damage inflicted on the parasitoid immature stages in the host compared to the control. A few mummies had large ragged-edged holes chewed into their dorsal or lateral surfaces, which suggests that some ant workers with their strong chewing mouthparts might have been responsible for the mummy predatory attempts. The importance of ant predation on parasitized insect species as a protein food source to complement their carbohydrate-rich diet has been widely documented [63,64,65,66]. The underlying ability of ant species to detect and selectively removed parasitized insects can be attributed in part to the alarm pheromones released by parasitized insects [67] or changes in the insect’s behavior when it is parasitized [68,69].

Many studies have shown that workers of more aggressive ant species often attack and kill adult female parasitoids [37,70]. *Anagyrus* species near *pseudococci* Girault (Hymenoptera: Encyrtidae) has been reported to suffer high direct mortality when exposed to *Anoplolepis steingroeveri* Forel (Hymenoptera: Formicidae), *Crematogaster peringueyi* Emery (Hymenoptera: Formicidae) and *Linepithema humile* (Mayr) (Hymenoptera: Formicidae) [37]. In the current study, *A. pseudococci* also suffered significantly high direct mortality due to encounters with *O. longinoda* workers, which led to a quick decline in parasitoid populations over time. The majority of the parasitoids in this study were seized and killed during the process of oviposition as they concentrated every effort on subduing the host rather than the reactions of the nearby intruding ants. This is similar to the observation made by Mgocheti and Addison [37], who reported that *Anagyrus* sp. were readily seized by ants when they got entangled with the host while ovipositing as they struggled to retract their ovipositor. This implies that the detrimental effect of *O. longinoda* workers on the parasitoid wasps will be more severe with increased ant populations considering that they are highly organized aggressive predators with extensive foraging activities throughout the areas occupied by their colonies, and they have a high potential to expand into new areas.

It is apparent that *O. longinoda* did not only interfere with the percentage of parasitism of their adopted *R. iceryoides*, but also reduced *A. pseudococci* abundance by causing direct mortality and low reproductive success. This is not unexpected because the negative effect of *O. longinoda* on other insect species reported by various authors [23,27,30,38,71] is also seen for mango mealybug parasitoids as presented here. It is important to mention here that the presence of the ant did not completely inhibit parasitoid landing and parasitism of *R. iceryoides* and it is possible that, with time, the parasitoid may be able to adapt to the presence of the ant and co-exist with it. However, while open-field studies are needed to validate our findings, future field releases of parasitoids to control mango mealybug infestation should be done with great caution taking into account the potential for biotic interference between the two natural enemies if both are recommended for use within the same agro-ecosystem.

Although we did not investigate the effect of semiochemicals produced by *O. longinoda* in the protection of *R. iceryoides* against parasitization by the parasitoid wasp, Appiah *et al.* [38] has clearly demonstrated that the presence of ant-produced semiochemicals on fruits and fruit trees both in the laboratory and opened field cages, respectively, had a negative effect on the searching behavior of parasitoids, thereby limiting their oviposition success on target hosts. This could be similar in the *O. longinoda* and *A. pseudococci* system leading to the evolution of avoidance behavior by the parasitoid at a distance from the aggressive ants, and this will further negatively impact on the efficiency of the parasitoids in the present study. Given that, in agricultural and natural ecosystems, *O. longinoda* obtains carbohydrate-rich honeydew from *R. iceryoides* that serves as a metabolic fuel for the ants, they will always exhibit protected behavioural dominance and promote the latter’s infestation to economically unacceptable levels. Therefore, mango growers should engage in orchard management practices that improve and conserve natural and augmentative released populations of this parasitoid species to suppress *R. iceryoides* populations. In this regard, ant control or reduced ant populations in orchards should be considered a priority when parasitoids are to be used as biological control agents of hemipteran pests as ant presence will not only affect parasitoid abundance but also reproductive success and possibly the oviposition strategy of female parasitoids.

## 5. Conclusions

Although, open-field studies are needed to validate our findings, ongoing field releases of parasitoids in different agro-ecosystems for pest control must take into account the potential negative effect of *O. longinoda* to minimize the occurrence of biotic interference. Whether the impact observed in the current study will affect co-existence of the two natural enemies in the field depends on several factors. Firstly, the concentration and composition of *O. longinoda* population on the different host plants with populations of the invasive mealybug *R. iceryoides* and how the parasitoid *A. pseudococci* respond to it will be crucial. Secondly, it is possible that the response of the parasitoid to ants attack may be a learned response that varies with the frequency of interactions between the parasitoid and the ant. Indeed, not all interactions between foragers and ant antagonists were necessarily fatal and in non-fatal encounters, the forager could benefit from learning the extent of the danger of such an interaction. Thirdly, the level of co-evolution between the natural enemies and their resident ant species as well as host plants may also contribute to variation in the strength of trait-mediated interactions.
